# Core defense hotspots within *Pseudomonas aeruginosa* are a consistent and rich source of anti-phage defense systems

**DOI:** 10.1093/nar/gkad317

**Published:** 2023-05-04

**Authors:** Matthew C Johnson, Eric Laderman, Erin Huiting, Chi Zhang, Alan Davidson, Joseph Bondy-Denomy

**Affiliations:** Department of Microbiology and Immunology, University of California, San Francisco, San Francisco, CA 94158, USA; Department of Microbiology and Immunology, University of California, San Francisco, San Francisco, CA 94158, USA; Department of Microbiology and Immunology, University of California, San Francisco, San Francisco, CA 94158, USA; Departments of Biochemistry and Molecular Genetics, University of Toronto, 661 University Ave, Toronto, ON M5G 1M1, Canada; Departments of Biochemistry and Molecular Genetics, University of Toronto, 661 University Ave, Toronto, ON M5G 1M1, Canada; Department of Microbiology and Immunology, University of California, San Francisco, San Francisco, CA 94158, USA; Quantitative Biosciences Institute, University of California, San Francisco, San Francisco, CA 94158, USA; Innovative Genomics Institute, Berkeley, CA 94720, USA

## Abstract

Bacteria use a diverse arsenal of anti-phage immune systems, including CRISPR-Cas and restriction enzymes. Recent advances in anti-phage system discovery and annotation tools have unearthed many unique systems, often encoded in horizontally transferred defense islands, which can be horizontally transferred. Here, we developed Hidden Markov Models (HMMs) for defense systems and queried microbial genomes on the NCBI database. Out of the 30 species with >200 completely sequenced genomes, our analysis found *Pseudomonas aeruginosa* exhibits the greatest diversity of anti-phage systems, as measured by Shannon entropy. Using network analysis to identify the common neighbors of anti-phage systems, we identified two core defense hotspot loci (cDHS1 and cDHS2). cDHS1 is up to 224 kb (median: 26 kb) with varied arrangements of more than 30 distinct immune systems across isolates, while cDHS2 has 24 distinct systems (median: 6 kb). Both cDHS regions are occupied in a majority of *P. aeruginosa* isolates. Most cDHS genes are of unknown function potentially representing new anti-phage systems, which we validated by identifying a novel anti-phage system (Shango) commonly encoded in cDHS1. Identifying core genes flanking immune islands could simplify immune system discovery and may represent popular landing spots for diverse MGEs carrying anti-phage systems.

## Graphical Abstract

**Graphical Abstract:**
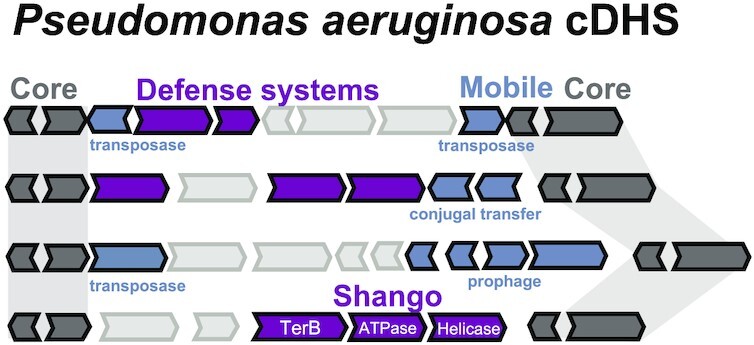
Core defense hotspots are regions of a bacterial genome where multiple anti-phage immune systems are encoded between core genes.

## INTRODUCTION

Bacteria are subjected to a diverse community of bacteriophages (phages) and rely on various anti-phage immune systems to block phage replication. Understanding the immune response to phage will be central to phage therapy success and likely new biological mechanisms await discovery. Anti-phage defense systems can be found adjacent to other defense systems within the same genomic locus in so-called ‘defense islands’ (1). Defense islands are a rich source of novel anti-phage systems (2,3) with some showing homology to human and plant immune systems (4). For example, a bacterial version of Gasdermin, an innate mammalian immune system that forms large pores in the cellular membrane, was recently discovered by searching for gene families enriched in defense islands (5). CBASS is another remarkable example of an anti-phage immune system found in defense islands, which is homologous to cGAS-STING innate immunity (6).

Defense islands are often found peppered across the genome due to being frequently encoded by mobile genetic elements (MGEs). Numerous reports have documented the phenomenon of the dissemination of mobile anti-phage immunity as a driver of phage resistance in the wild. For example, isolates of *Vibrio lentus* and *Vibrio cholerae* have MGE-disseminated islands that contain multiple anti-phage systems in hotspots to protect from phage (7,8). Additionally, *Escherichia coli* P2-like and P4-like prophages encode several anti-phage systems at a single hotspot that antagonize competing phage (9). These discoveries enable the facile identification of immune systems in these MGEs and in genomes where these MGEs are found but the complete identification of immune systems in a genome or species remains an important computational and experimental challenge.

Here, we sought to comprehensively identify and annotate the defense systems of *Pseudomonas aeruginosa*, a generalist microbe, opportunistic human pathogen, and a model organism for phage-host interactions and CRISPR-Cas biology. These efforts lead to the remarkable observations of two conserved loci in most *P. aeruginosa* genomes that serve as hotspots for immune systems, which we call core defense hotspots (cDHS). We show that cDHS1 contains at least 31 different immune systems across >1600 isolates, with many genes of unknown function likely involved in phage defense. One operon that is commonly present in cDHS1 (a new three gene system we call ‘Shango’, containing TerB, helicase, and ATPase domains) inhibited replication of several phage families. In contrast to defense islands encoded on variable MGEs, this core hotspot is encoded in the same region of the host chromosome in all isolates and appears to have been assembled by numerous diverse MGEs. We also find another conserved hotspot with extensive immune diversification called cDHS2, which has at least 24 different immune systems. These regions are likely rich in new anti-phage systems and do not require any specific existing immune system or single MGE family to find them. Core defense hotspots might be a common phenomenon in bacteria and network analyses like ours provide a road map to their discovery.

## MATERIALS AND METHODS

### Finding core defense hotspots in *P. aeruginosa*

To identify anti-phage systems with high confidence and subsequent retrieval of defense islands in *P. aeruginosa*, we curated several anti-phage proteins and created Hidden Markov Models (HMMs) for them. HMMs were created by taking a set of seed protein sequences, removing all redundant sequences, and clustering them using MMseqs2 easy-cluster at high sensitivity (*-s 7.5*). A multiple sequence alignment from each cluster with more than five members was then created using Clustal Omega, and HMM models were built using hmmer (hmmer.org). HMMs for CRISPR-Cas systems were obtained from CRISPRCasFinder and matches to these HMMs were searched for using macsyfinder (10,11). Representative sequences for immune systems from BREX, DISARM, Gabija, Wadjet, Septu, Shedu, Thoeris, Druantia, Lamassu, Hachiman, Kiwa, Zorya, CBASS, Retrons, etc., were obtained from literature (2,3,12,13). Representative sequences for Type I, II, III, and IV RM systems were obtained from REBASE’s gold standard collection (14). Type II and IV RM systems were searched for by BLAST as effective HMMs cannot be built for these systems (15,16). The sequences for each gene in an experimentally verified system consisting of at least two genes were PSI-BLASTed against all genomes available on NCBI in GPFF format (12/03/2020) with four iterations and an inclusion e-value threshold of 5.0 × 10^−3^. Systems were identified by searching for hits (*e*-value < 1.0 × 10^−10^) of each gene that were directly adjacent to a hit corresponding to its neighbor in the system it is from and part of a locus where all genes from a given system were accounted for. HMMs for all genes from each system were created from the PSI-BLAST hits that were determined to be part of a *bona fide* system using the method described above.

Immune systems were found by searching for all genes comprising that immune system using hmmsearch or BLAST (only if an HMM could not be built for that gene). Searches were performed against databases in gembase format. The database was first clustered with mmseqs2 easy-linclust (linear clustering time) with an identity cutoff of 0.9 (17). Only representative sequences for a cluster were included in the reduced database. All genes for systems of interest were searched for in the reduced database using hmmsearch or BLASTP as appropriate. If a protein in the reduced database was found to be a hit for a gene in a system of interest, all other proteins in the original database that belong to the same cluster as that protein were considered hits for that gene. Once hits for all genes of interest were identified, systems were identified. By using representative sequences, the computational burden was greatly reduced. Information of the member sequences were kept by storing the index in the genome (Supplementary Figure S1A). A system is called if it contains all essential genes or enough accessory genes to reach a minimum threshold. HMMs and system definition parameters can be found at https://doi.org/10.5281/zenodo.7754202.

### Network analysis

For each anti-phage system identified, the genes spanning 5 kb upstream and downstream of the system were selected (10 kb window surrounding system). A bipartite network was created by treating the anti-phage system as a system node and the neighbor genes as neighbor nodes. The edges between system and all neighbor nodes were established and if neighbors were found frequently to the same system in different loci, the edge weights would increase. This was repeated for every anti-phage system and its locus to create a large bipartite network. To remove redundant neighbor nodes, the protein sequences of neighbors were clustered using MMseqs2 easy-linclust. If nodes belong to the same protein family, they become a single node, keeping all edge connections of the members. Cytoscape 3.9.1 (18) was used to visualize the resulting network.

### cDHS1 and cDHS2 region analysis

To identify the cDHS1 region in different isolates, two conserved genes were chosen for their conservation in *Pseudomonas*. The protein sequences of the MerR transcriptional regulator (PA14_RS11700) and the DUF4011 protein (PA14_RS11765) of PA14 were used as markers of the left and right flanking core genes respectively. MMseqs2 search function was used to find orthologs in our database downloaded from www.pseudomonas.com. Contigs were filtered for having both markers. Data on these contigs and their hotspot can be found in supplementary Table S1. cDHS2 locus was retrieved by using the tmRNA site (ssrA; 4749731..4750083) and the VOC (PA14_RS21865), LysR (PA14_RS21870), RidA (PA14_RS21880) like proteins as markers. While our custom anti-phage annotation pipeline was used as a preemptive tool that led us to the discovery these two regions, Defense Finder was employed to annotate the immune systems present in both cDHS1 and cDHS2 because of its more complete anti-phage database. Files needed to run our anti-phage annotation tool can be found at https://doi.org/10.5281/zenodo.7754202.

### Phylogenetics of cDHS1

To construct a phylogeny of the *P. aeruginosa* isolates, the redundant nucleotide sequences of cDHS1 were removed using MMseqs2 and the entire genome for the remaining set was retrieved. Gubbins (https://github.com/nickjcroucher/gubbins) was used to construct a RAxML maximum likelihood tree. Gubbins was used because it accounts for extensive horizontal transfer by removing hypervariable regions before alignments. iTOL (https://itol.embl.de) was used to apply features of the cDHS1 to the tree.

To identify long range synteny, tree branch distances were calculated between every pairwise isolate using the Phylo package from BioPython. The cDHS1 nucleotide MASH-distance was calculated between every pairwise isolate using MASH (https://github.com/marbl/Mash). Long range synteny was defined as isolated having an evolutionary tree distance above 0.35 and an cDHS1 MASH-distance above 0.9. Occurrences of rapid diversification were defined as isolates having a tree distance below 0.05 and a cDHS1 MASH-distance above 0.38.

### Domain analysis

To better understand the molecular function of the genes encoded at these hotspots, domain analysis was performed on all protein-coding genes within the left and right flanking core genes. Pfam domain models were obtained from Conserved Domain Database (3 May 2022) and searched against the pool of proteins within cDHSs that were not annotated by Defense Finder as belonging to an immune system. Rpsblast (2.11.0+) using an e-value cutoff of 0.01 was used to search for the pfam models.

### Mobilome analysis

MobileOG-db was used to quantify mobile elements in cDHSs (19). Sites were separated into three categories (integrative, prophage or conjugative) if they matched ‘integration’, ‘phage’, ‘conjugation’ in their major category annotation.

Intact conjugation systems were identified using macsyfinder CONJscan module that identifies 8 types of conjugation systems (MPF B, C, F, FA, FATA, G, I, T). https://github.com/gem-pasteur/Macsyfinder_models/tree/master/models/Conjugation.

### Strain and plasmid construction

cDHS1 of PA14 contains Shedu (PA14_RS11720), a large multi-domain helicase (PA14_RS11730), a three-gene TerB-like protein (PA14_RS11735), ATPase (PA14_RS11740), and DEAD/DEAH box helicase (PA14_RS11745) system, referred to as Shango. The three gene system including its native promoter with ihfA and MerR genes found in the flanking regions of cDHS1 (Figure 3A) was cloned in pHERD30T digested with PstI-HF and SacI-HF enzymes for 1 h at 37°C. PCR was used to amplify the system from PA14 and subsequently assembled in digested pHERD30T using Gibson assembly (pMJ31). Phage sensitive host PAO1 was chosen because it is sensitive to several phages, allowing us to screen a panel of strongly lytic phage to identify phage resistant phenotypes more clearly. pMJ31 was electroporated into PAO1 and recovered with SOC for 1 h and plated on gentamicin 50 ng/μl.

### Phage assays

Phages were 10-fold serial diluted 8 times in SM phage buffer. 2 μl of the phage dilutions were plated on PAO1 either containing pHERD30T empty vector or pMJ31 (pHERD30T-Shango) that were 150 μl diluted into 3 ml of 0.7% top agar. Plates were left to dry under a flame for 10 min and incubated upside down, to prevent condensation, at 37°C overnight. Plate images were taken with a Biorad Gel Doc EZ imager. EOP was calculated as a ratio of the number of plaque forming units (PFUs) that formed on the strain expressing Shango divided by the number of PFUs that formed on the strain with an empty vector. Liquid culture phage infections were prepared with strains grown overnight and diluted 100× in LB supplemented with 10mM MgSO4, gentamicin, and 0.3% arabinose. 140 μl of bacterial culture was infected with 10 μl of serially diluted phage in a 96-well plate. Growth was monitored over 35 hours using a Synergy H1 microplate reader (BioTek) shaking at 37°C. Assay was done in three replicates. Data shown represents a consensus phenotype.

## RESULTS

### Immune system detection

To enable the identification of anti-phage bacterial immune systems, we developed a bioinformatic pipeline to annotate anti-phage systems. We used a combination of hidden Markov models (HMMs) and BLASTp to annotate immune systems in large bacterial genome databases (Supplementary Figure S1A). To test the annotation accuracy of our models and approach, we measured our pipeline's ability to identify the systems included in Doron *et al.* We included only the genomes that were found to have at least one system in Doron *et al.* and were available from NCBI (14 296 genomes). Our models had very high sensitivity and specificity, and generally low false discovery rates (Supplementary Figure S1B).

We then applied our approach to all bacterial genomes available on NCBI as of 12/03/2020 (184541 genomes). Across all species we found that the most common systems were RM systems with >70% of all genomes surveyed containing at least one RM system. The three next most common systems were CRISPR-Cas, CBASS, and Gabija, which were found in 35%, 16% and 10% of genomes, respectively (Supplementary Figure S2). Our results on overall immune system abundance are qualitatively similar to those recently presented in Tesson et al., 2022, however our database contained models for fewer systems.

### *Pseudomonas aeruginosa* as a model for immune system discovery and gene organization

**Figure 1.**
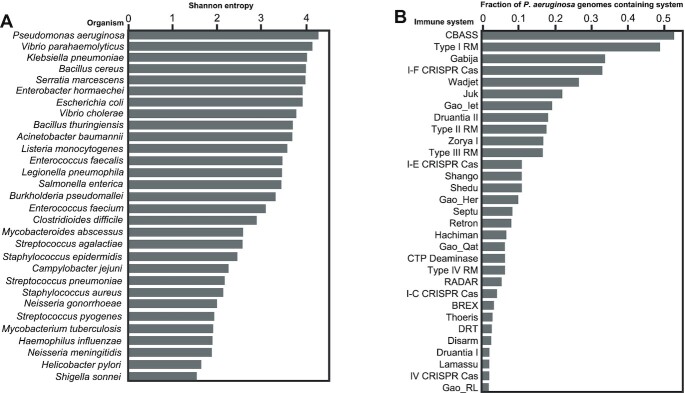
Anti-phage diversity and distribution. (**A**) Shannon entropy as an indicator of immune system diversity across bacterial species with at least 500 genomes in NCBI. (**B**) The fraction of *P. aeruginosa* genomes that contain each of the antiphage systems shown. Only anti-phage systems present in at least one percent of *P. aeruginosa* genomes were shown above. Juk stands for "Jumbophage killer" and was first identified in *P. aeruginosa* strain PA14.

Shannon entropy is a measure of uncertainty or randomness of a system. Here, we use Shannon entropy of defense systems as a proxy for defense diversity. To calculate Shannon entropy for a species defense repertoire (species with fewer than 200 genomes deposited in NCBI were removed from analysis) we calculated the entropy of defense as the negative sum of *p*_sys_*log_2_(*p*_sys_) for each system present in the species. *p*_sys_ is the ratio of the frequency of a given system compared all systems in a species. For example, if a species has 10 different defense systems and a specific system is present in the species 3 times, the *p*_sys_ of that system would be 0.3. We found that *P. aeruginosa* has the highest Shannon Entropy of defense, indicating that it's extremely rich with immune systems (Figure 1A). Other bacterial species with highly diverse immune arsenals include *Escherichia coli, Klebsiella pneumoniae* and *Vibrio parahaemolyticus*. On the other hand, *Campylobacter jejuni, Heliobacter pylori* and *Neisseria meningitidis* all have immune arsenals which are not nearly as diverse as measured by Shannon entropy. To further assess the richness of anti-phage systems across different bacterial genera, we used Defense Finder to create rarefaction curves of the number of unique anti-phage systems as a function of number of genomes analysed (Supplementary Figure S3). The results corroborate with the results from the Shannon entropy analysis. On the Genus level, *Vibrio*, *Pseudomonas*, *Klebsiella* and *Escherichia* are highly rich in anti-phage systems. The immune system prevalence and diversity in *P. aeruginosa* coupled with genetic tractability and a diverse isolated phage population suggests that *P. aeruginosa* will be a good model organism for the characterization and discovery of new anti-phage immune mechanisms.

In *P. aeruginosa*, CBASS and Type I RM are the most common anti-phage systems, present in 53% and 49% of genomes respectively. Gabija, Type I-F CRISPR-Cas, Wadjet and Jumbophage killer (Juk) are the next most common systems, and all are present in >20% of *P. aeruginosa* genomes (Figure 1B). Furthermore, of the ten most common anti-phage systems in *P. aeruginosa* only four (Type I and II R-M, Type I-F and I-E CRISPR-Cas) had been known before 2018, suggesting that there are many antiphage systems waiting to be discovered.

### Core defense hotspot 1 (cDHS1) in *P. aeruginosa*

**Figure 2.**
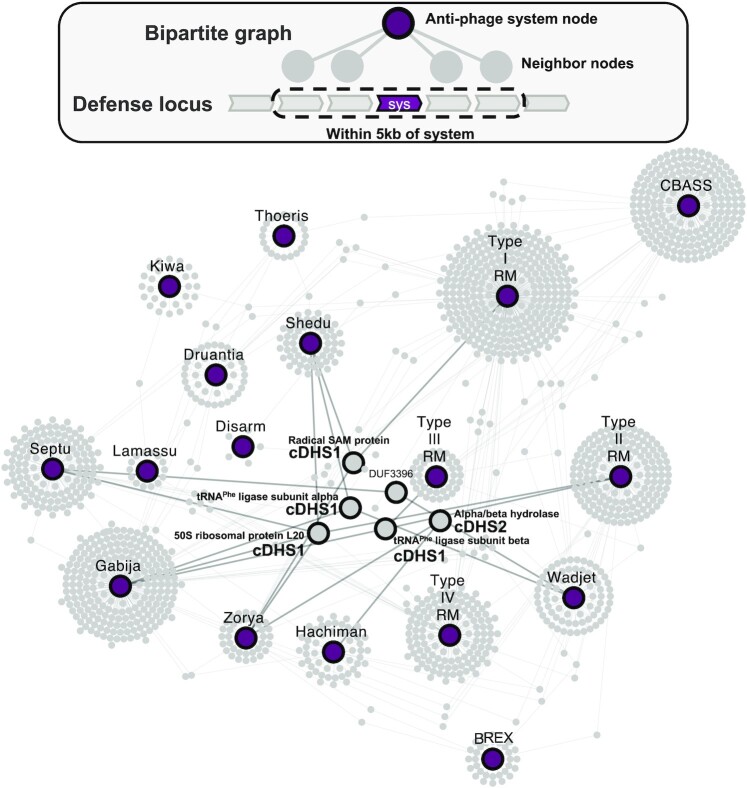
Defense island association network. Purple nodes represent anti-phage systems and gray nodes represent genomic neighbors of that system within 5 kb upstream and downstream (total 10 kb window). Most gray nodes are only represented nearby one system and are those shown acting as a ‘halo’ around the purple nodes. Other nodes are present nearby multiple systems and are towards the center of the network. The large gray nodes with black borders are nodes of interest because they represent genes that are core/conserved and associated with many different immune systems.

After validating the accuracy to annotate immune systems with our pipeline, it was applied to the *Pseudomonas* database (www.pseudomonas.com) containing at least 4640 *P. aeruginosa* genomes. Specifically, our goal was to understand the complement of anti-phage systems in *P. aeruginosa* and where in the genome they are encoded. The genes found within 5 kb upstream and downstream of a known immune system (i.e. putative defense islands or genes flanking a defense island) were retrieved from the output and encoded as a network (Figure 2). This resulted in a large, bipartite network with most nodes having a degree of one (gene neighbors only found nearby a single known immune system) and several nodes having a high degree (genes associated to several immune system). We considered that nodes with the highest degree might be candidate immune systems. This was not the case however, as we observed a clear cluster of gene nodes with functions related to translation: Phenylalanine tRNA ligase subunit alpha (PheS), Phenylalanine tRNA ligase (PheT) subunit beta and 50S ribosomal L20 (rpIT) were associated with nine different immune systems (Figure 2). *pheT* is essential in *P. aeruginosa* and broadly conserved (20). This region also contained a MerR family transcriptional regulator, an integration host factor gene, ihfA, and a proline tRNA.

**Figure 3.**
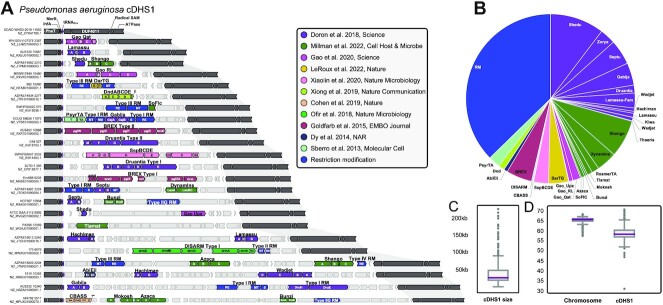
Core defense hotspot 1 (cDHS1). (**A**) Representative isolates with cDHS1 chosen to show the diversity in anti-phage systems. Dark gray genes are marker genes that define the boundaries of the hotspot. Light gray genes indicate non-defense genes and are neighbors to the defense system which are colored based on the publication they were discovered in. (**B**) Pie chart of the different immune systems found in cDHS1, colored by publication. (**C**) Size distribution of cDHS1 in kb. (**D**) GC% difference between the host chromosome and the defense hotspot.

These genes related to translation appeared as the left flank to a locus that contained numerous immune systems (Figure 3A), with the right flank containing a conserved small hypothetical gene, a large DUF4011 gene, and a radical SAM protein which was also a node of interest in our network (Figure 2). To get a sense for how conserved this locus is in *P. aeruginosa*, homologs of the *merR* gene (left flank) and the DUF4011-encoding gene (right flank) were searched across the *P. aeruginosa* genome database. These flanking core genes were found in every *P. aeruginosa* isolate. We therefore consider this region to be a defense hotspot, which we dub as core defense hotspot 1 (cDHS1). cDHS1 is marked by consistent flanking core genes and not obviously a member of any one group of mobile genetic elements (MGEs). Genomes with both flanks on the same contig were filtered and resulted in more than 1600 total cDHS1 regions. cDHS1 ranged dramatically in size (1–224kb with a median of 26 kb, Figure 3C). %GC content is often a signature to distinguish between the core and horizontally acquired genes in a species. Integrated elements, MGEs, and prophages usually have a lower GC% compared to the host chromosome. We measured the cDHS1 region as having a lower GC% content compared to the *P. aeruginosa* genome suggesting a more recent acquisition (58.9% versus 65.3%) (Figure 3D).

Defense Finder, a recently published anti-phage annotation tool (21) was used to annotate the immune content of this locus as it contains the most comprehensive set of immune system HMM profiles to date and is orthogonal to our own anti-phage annotation tool used to initially identify this locus. Across all cDHS1 regions, Defense Finder annotated 31 different immune systems, a remarkable hotspot for anti-phage immunity. The ubiquitous nature of RM across prokaryotic genomes persists in cDHS1. Of the total number of defense systems in cDHS1, Shedu, Type I RM and Type III RM were the most common immune systems found, with over one third belonging to RM (Figure 3B). We find that another large percentage of immune systems come from recent publications that used computational analysis of defense islands (2,3,22) (Figure 3B). We find at least one immune system in 83.7% of cDHS1 loci (Supplemental Table S1), clearly demonstrating an immune functionality for cDHS1. While this is a strong anti-phage hotspot, 87% of genes in the cDHS1 locus are not a part of a known immune system, suggesting this locus will serve as a rich source of novel discovery.

### Core defense hotspot 2 (cDHS2) in *P. aeruginosa*

**Figure 4.**
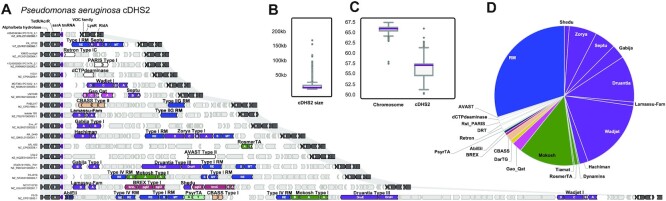
Core defense hotspot 2 (cDHS2). (**A**) Representative isolates with cDHS2 showing diversity in anti-phage systems (same color scheme as Figure 3) (**B**) Size distribution of cDHS1 in kb. (**C**) GC% difference between the host chromosome and the defense hotspot. (**D**) Pie chart of the different immune systems found in cDHS2 (same color scheme as Figure 3).

Since the network revealed cDHS1 through high degree nodes, we asked if other high degree nodes would point to another hotspot. We noticed an alpha/beta hydrolase annotated node that pointed us to another defense rich locus in *P. aeruginosa*. We performed the same analysis for this locus as we did for cDHS1 and found it to exhibit features like cDHS1, we denote this site cDHS2 (Figure 4A). cDHS2 contains 24 different immune systems, less than that of cDHS1, but three systems were present in cDHS2 that were not in cDHS1 (Retron, PARIS, and dCTP deaminase). It showed clear hypervariability and diversification with a range in size between 0.3 kb and 168 kb (Figure 4B). It also has a different composition of immune systems. While restriction modification is the chief system at both cDHS1 and cDHS2, almost half of the systems found at cDHS2 are from Doron *et al.* Druantia and Wadjet account for roughly 25% of the immune systems within cDHS2, while these systems are rare (<2%) in cDHS1. Mokosh is also frequent in cDHS2 but virtually absent from cDHS1. Conversely, Shango and BREX make up ∼12% of systems in cDHS1 but Shango is not found and only two occurrences of BREX (<1%) in cDHS2. Similar to cDHS1, the cDHS2 site is flanked by a non-coding RNA, in this case an *ssrA* transfer-messenger RNA. An immune system is found in 15.2% of cDHS2 sites, far less than that of cDHS1. The right flanking region contains genes with VRR-NUC domains, NUDIX domains, VOC family and LysR family transcriptional regulator as well as a variety of other small hypothetical proteins.

### Domain analysis

**Figure 5.**
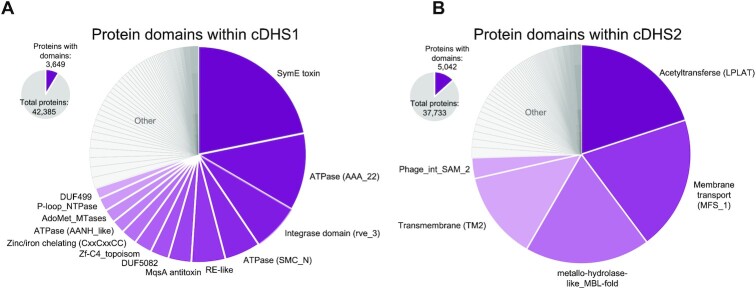
Domain analysis of cDHS1 and cDHS2. Pie chart summarizing the non-defense genes in each hotspot. Note that most genes do not match a pfam domain. Of the genes with domains, the most common are indicated in purple for cDHS1 (**A**) and cDHS2 (**B**).

To understand what other genes might be in these hotspots and to search for potential mobilizing genes, we performed Pfam domain analysis on all the genes not annotated by our pipeline or Defense Finder. While most genes are hypothetical and have no known domains, we find many domains related to those found in anti-phage defense systems (toxins, helicases, ATPases, etc.) (Figure 5A). A *symE* toxin is commonly found among cDHS1 sites. *symE* is small toxin belonging to the Type I toxin-antitoxin systems characterized in *E. coli*, the cognate antitoxin is an antisense RNA that binds to the 5′ untranslated region (UTR) of *symE* (23,24). This TA module could be an anti-phage defense system or perhaps acting an addiction module, stabilizing the presence of the locus. Domain analysis of cDHS2 paints a different story. Most genes (87%) contained no known pfam domains, but of those that had domains, many of them matched domains that belong to conjugative systems (Figure 5B). Deeper analysis specifically looking for intact conjugative systems identified several full conjugative systems. (Supplemental Table S1).

### Shango is an anti-phage system

**Figure 6.**
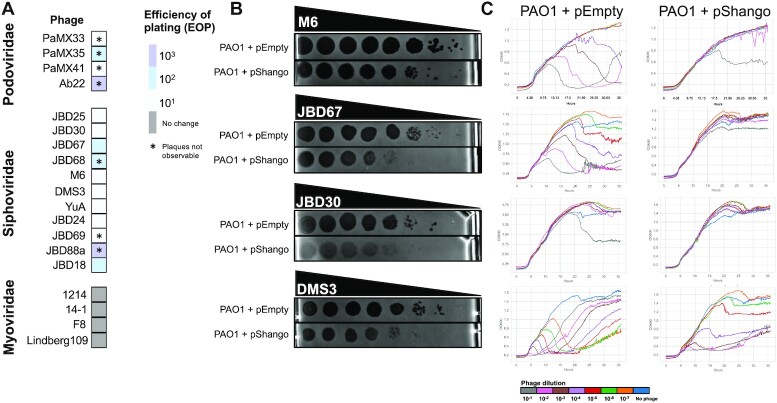
Shango phage assays. (**A**) A heatmap of efficiency of plating (EOP) of several different Pseudomonas phage on a strain. EOP is calculated as the ratio of the number of plaques between the PAO1 + pEmpty (empty vector) strain and the PAO1 + pShango expressing strain. Phage are grouped by their morphology type (Myovirus, Siphovirus, and Podovirus). (**B**) Ten-fold dilutions of phage lysates (spotted left to right) on a lawn of PAO1 + pEmpty or PAO1 + pShango. (**C**) The same strains and phages were used in liquid infection, where OD600 is plotted to quantify culture lysis due to phage replication. Phage dilutions are labeled at the bottom. Three replicates were performed for liquid and plate assays. Single replicates shown represents the consensus of all three.

We noticed a three gene system common in cDHS1, including model strain PA14. This system contains a TerB-like protein, a helicase, and an ATPase. We expressed this system in strain PAO1 (a strain with a small cDHS1 encoding only a Type I R-M system) and performed phage plaque assays with several phages from different families in liquid and solid media (Figure 6). We observed modest (i.e. 10–100-fold EOP change) anti-phage activity against many phages on solid media, (Figure 6B). For several phages, we noticed a reduction in plaque size and/or clarity. Notably, this was not observed for all phages titrated on lawns expressing Shango (Supplementary Figure S4). Generally, we see strong protection of cell cultures from phage-induced lysis over a broad range of phage concentrations during liquid growth (Figure 6C, Supplementary Figure S5). We call this anti-phage system, Shango. Shango was recently identified in immune islands in *E. coli* and modestly inhibited the replication (i.e. 10-fold) of two different phages (22).

### Core DHS1 undergoes rapid immune adaptation

**Figure 7.**
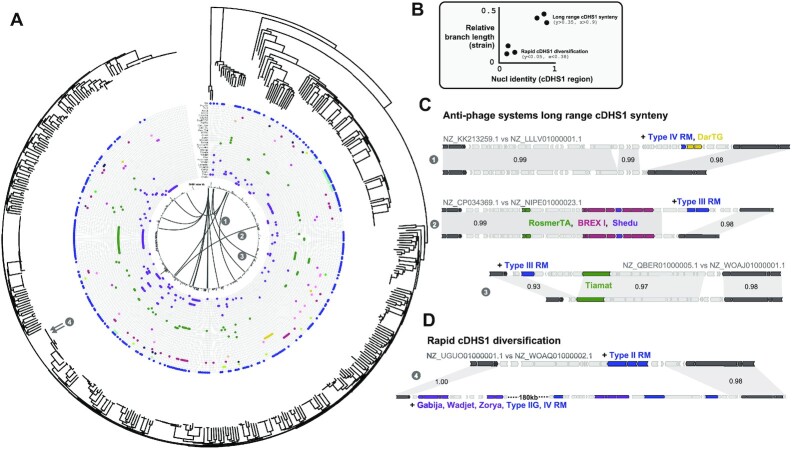
Phylogeny and synteny of cDHS1. (**A**) A RAxML tree of the isolates encoding cDHS1 with the systems within the hotspot indicated by a colored dot (same color scheme as Figure 3). cDHS1 size is represented by the black bars in the center of the tree. Lines between pairs of isolates indicate long range synteny which is exemplified on the right side. (**B**) Example plot showing how long range synteny and rapid diversification is determined. Pairs of isolates with high branch length between their points on the tree (distant isolates) and high nucleotide identity of cDHS1 indicate long range synteny. Pairs with low branch lengths (close isolates) but high cDHS1 identity indicate rapid diversification. (**C**) Anti-phage systems long range synteny with the same color scheme as Figure 3. Light gray ribbons represent high nucleotide identity and the values inside the ribbons represent the exact nucleotide identity. (**D**) Rapid cDHS1 diversification where two isolates with very close proximity on the tree have extremely different cDHS1 compositions.

To understand the cDHS1 immune diversification across *P. aeruginosa* isolates, a whole genome phylogeny was constructed of all isolates containing an intact cDHS1 (Figure 7A). Evolutionary discordance between the chromosome and cDHS1 is expected if these sites are horizontally acquired (Figure 7B). Indeed, we see this discordance amongst cDHS1 sites in the tree, where isolates in distant clades of the tree have high cDHS1 similarity at the nucleotide level (Figure 7C). While isolates that are very closely related tend to have similar cDHS1 sites, occasionally there is rapid diversification of the hotspot (Figure 7D) that can gain several kilobases. These data confirm what is expected given the diversity of systems at this single locus, that this region is likely mobilized and diversified through horizontal gene transfer. One potential route would be generalized phage transduction and recombination since the regions flanking cDHS1 are highly conserved in all isolates.

### Mobilome of cDHS

**Figure 8.**
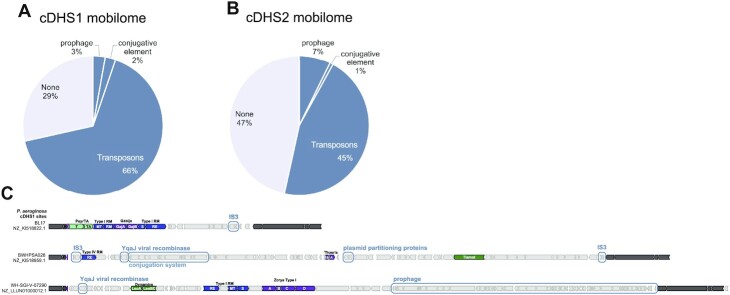
Mobilome of cDHS. Pie chart of the mobile element types that make up cDHS1 (**A**) and cDHS2 (**B**). (**C**) Orf maps of cDHS1 that carry mobile genes (boxed). Defense systems are colored based on Figure 3.

We next assessed the mobilome within genes between the cDHS flanking regions and found no single MGE type amongst the sites, rather a combination of transposons, conjugative elements, and prophages were identified. 29% of cDHS1 and 47% of cDHS2 sites had no detectable mobile gene (Figure 8A, B). Transposases were the most found mobilizing gene in both hotspots, most of them belonging to the IS3 family (Supplemental Table S1). Most transposases were found at the boundaries of the hotspots (Figure 8C). Prophages (full or partial) were found in a small percentage of cDHS1/2 sites as well as genes of viral origin, like the YgaJ viral recombinase. We also find examples of type IV conjugation systems and plasmid partitioning proteins in a small fraction of cDHS sites. Fully intact conjugation systems were infrequent but identified sporadically. cDHS1 contained intact T4SS Type mating pair formation T (MPF_T_) infrequently. cDHS2 also carried MPF_T_ conjugative systems as well as MPF_G_. The ICEclc element found in several *Pseudomonas* species is type MPF_G_ and MPF_T_ are often found with flanking tRNA sites (25). The ability of these sites to accept a wide range of MGEs could be why cDHS are almost always occupied. Notably, the flanking ‘core’ region of both cDHS1 and 2 possess non-coding RNA genes (proline tRNA or tmRNA *ssrA*) which are popular landing sites for MGEs (26–29). cDHS sites therefore apparently act as a landing pad for mobilization events stemming from diverse MGEs encoding anti-phage immune systems.

## DISCUSSION

The recent discovery of numerous anti-phage immune systems in bacterial genomes has led to an examination of their genomic distribution and location. Fascinating examples of MGE transferred hotspots of anti-phage immunity have been documented that control ecological outcomes during phage-host interactions in *Vibrio* and *E. coli*, for example (7–9,30–32). In contrast to these phage defense MGEs, we show that core defense hotspots with conserved genomic markers flanking immunity regions can be predictive of an immune locus, without an obvious single mobilizing mechanism. While immunity carrying MGEs may integrate in some genomes but not others, cDHS regions are essentially always occupied in all isolates. This differentiation aligns with the reports of two types of flexible islands in clonal prokaryotes, replacement and additive islands. Replacement islands, like the hotspots reported by others in in *Vibrio* and *E. coli*, are made up of short defense cassettes that share no sequence homology to each other. Additive islands, like cDHS, result from multiple discrete integration and are often associated with tRNAs (33). An outstanding question not addressed here is whether cDHS regions are common in other bacterial clades, which will be addressed in future work.

cDHS regions in *P. aeruginosa* carry a large variety of mobile genes, have a large size variance across isolates, and can likely be horizontally transferred as we observe distant isolates carrying almost identical cDHS content (Figure 7C). Additionally, cDHS1 often possesses at least one immune system somewhere in the locus. Previously described hotspots for anti-phage immunity, for example the elements present at 41 mobile defense hotspot sites described in *E. coli*, typically are less occupied (each locus is occupied in 8% of isolates on average), have fewer total systems per locus, and contain a single MGE type (32). These are signatures of a landing spot for a specific MGE compared to the several mobile elements of cDHS. A plausible three-step model for cDHS regions could be at play where (i) cDHS contains a target site that acts to seed the initial insertion element that could (ii) carry additional target sites which facilitate subsequent insertion events and then (iii) dissemination of the entire hotspot to other isolates. If the founding MGE that recognizes the target site bears an anti-phage system, it may stage the region with a bias to maintaining additional defense systems as is the case for defense islands (1). If the target site is disrupted after the initial integration event, only MGEs with target sites near or within defense systems would be compatible with the region thereafter. Finally, the entire region has potential to laterally transfer due to the conserved flanking regions through generalized phage transduction and homologous recombination, as this would not leave MGE-derived scars in the genome. The collective immune system repertoire at these sites could also then be regulated in a coordinated way. How these enigmatic core defense hotspots elements are built and regulated, and whether they can transfer in this manner will be important areas for future work.

The identification of cDHS greatly simplifies the identification of known and new immune systems in *P. aeruginosa*. Notably, we show the utility of immune discovery via cDHS regions by identifying Shango from the cDHS1 of PA14 which is an anti-phage system with a TerB-like domain. It was previously hypothesized that the widespread nature of tellurite resistance (Ter) domains and its association to several anti-phage-like domains could be indicative of anti-phage mechanisms (34). Core defense hotspots identified here are present in nearly all strains of *P. aeruginosa* and > 80% of the time have at least one known immune system and likely many others that await identification. During the preparation of this manuscript, another report with 21 new immune systems was published, including Shango (22). The systems in that paper account for roughly a quarter of the known immune systems in cDHS1 and cDHS2. Together, the cDHS regions identified here are rich in immunity and greatly simplify the identification of immune islands in *P. aeruginosa*. Large immune islands likely present a rapid way for a strain to acquire a collection of new phage defenses at once.

## DATA AVAILABILITY

Code used to identify anti-phage systems as well as their HMMs can be found at https://doi.org/10.5281/zenodo.7754202.

## Supplementary Material

gkad317_Supplemental_Files
