# Supplementary material for: Core defense hotspots within Pseudomonas aeruginosa are a consistent and rich source of anti-phage defense systems
Source: Nucleic Acids Res. 2023 May 4;51(10):4995–5005. doi: 10.1093/nar/gkad317 (PMC10250203; doi:10.1093/nar/gkad317)
Supplement: gkad317_Supplemental_Files [file gkad317_supplemental_files.zip › supplemental_figures_legends.docx]

**Figure S1: Workflow for system annotation and performance.** (A) The ISLAND workflow requires building HMMs for genes in anti-phage systems, searching a pre-clustered protein database, and applying pre-defined logic. Models for genes in a system of interest are created by obtaining protein sequences from the literature corresponding to this system, clustering them, and then building HMMs from the clusters with at least three genes. Then systems are searched for in an ordered protein database (gembase format). (B) The sensitivity/specificity and false discovery rate of ISLAND of the Doron et al., 2018 systems.

**Figure S2: The abundance of each antiphage system in all genomes with anti-phage annotation tool**. This figure shows the fraction of all the genomes that harbor at least one representative of a given antiphage system using our in-house anti-phage annotation tool. The genome database was downloaded from NCBI on 12/03/2020 (184,541 genomes).

**Figure S3: Rarefaction Curve of Anti-phage Systems Across Different Bacterial Species.** The y-axis shows the number of unique anti-phage systems discovered as a function of the number of genomes analyzed. Data points represent different bacterial species, with black lines indicating different species of interest. The analysis was based on random subsamples of the data. A higher curve indicates greater diversity of anti-phage systems within the bacterial population.

**Figure S4**: **Shango phage assay.** Several phage plated on PAO1::EV and PAO1::Shango strains. Three replicates were performed, single replicates shown represents the consensus of all three.

**Figure S5: Growth curve assay.** Liquid infection assay with PAO1::EV and PAO1::Shango infected with phage. Strains were grown in a plate reader with OD600 tracked over time.
